# Genistein in autoimmune diseases: from experimental evidence to emerging clinical data

**DOI:** 10.3389/fimmu.2026.1890581

**Published:** 2026-07-06

**Authors:** Stefan Tukaj

**Affiliations:** Laboratory of Cellular and Molecular Immunology, Department of Molecular Biology, Faculty of Biology, University of Gdansk, Gdansk, Poland

**Keywords:** autoimmune diseases, genistein, immunomodulation, inflammation, phytoestrogens, translational immunology

## Abstract

Autoimmune diseases are chronic inflammatory disorders characterized by loss of immune tolerance, activation of autoreactive immune cells, and progressive tissue injury. Despite advances in immunosuppressive and biologic therapies, treatment responses remain heterogeneous and long-term therapy is frequently associated with adverse effects and economic burden. These limitations have stimulated increasing interest in complementary immunomodulatory strategies, including naturally occurring bioactive compounds. Genistein, a soy-derived isoflavone belonging to the phytoestrogen family, has emerged as a promising candidate due to its pleiotropic anti-inflammatory, antioxidant, and immunoregulatory properties. At the molecular level, genistein functions as a tyrosine kinase inhibitor and modulates several signaling pathways implicated in immune regulation, including NF-κB, MAPK, and estrogen receptor-dependent pathways. Through these mechanisms, genistein influences cytokine production, lymphocyte activation, T-cell differentiation, and cellular redox homeostasis. A body of preclinical evidence supports the immunomodulatory potential of genistein in multiple autoimmune disease models, including systemic lupus erythematosus, rheumatoid arthritis, experimental autoimmune encephalomyelitis, type 1 diabetes, Sjögren’s syndrome, autoimmune thyroid disease, and autoimmune blistering skin disorders. In these models, genistein administration has been associated with attenuation of inflammatory responses, reduced disease severity, and modulation of autoimmune pathways. Although precision medicine represents a major contemporary direction in autoimmune disease therapeutics, many patients continue to experience suboptimal clinical responses. In this context, multi-target compounds such as genistein may represent a potential adjunctive therapeutic approach. Clinical evidence remains limited; however, preliminary studies suggest potential immunomodulatory effects in humans. This mini review summarizes mechanistic, preclinical, and emerging clinical evidence regarding the role of genistein in autoimmune diseases.

## 1 Introduction

Autoimmune diseases represent a heterogeneous group of chronic disorders driven by a breakdown of immune tolerance to self-antigens, which results in persistent inflammation and progressive tissue injury. Taken together, they affect a considerable proportion of the global population, with epidemiological data suggesting that several percent of individuals may be affected in population-based studies (1). This broad category encompasses conditions such as rheumatoid arthritis (RA), systemic lupus erythematosus (SLE), multiple sclerosis (MS), autoimmune thyroid diseases, and rare autoimmune blistering skin diseases (AIBDs). Although clinically diverse, these disorders share core immunopathogenic features, including the activation of autoreactive lymphocytes and a dysregulated cytokine milieu that sustains chronic inflammation (2). Despite major advances in immunosuppressive agents and biologic therapies, current treatment strategies remain imperfect. Their clinical efficacy is often variable, and their long-term use is limited by high costs and clinically relevant safety concerns, including an increased susceptibility to infections and, in some cases, malignancies associated with prolonged immunosuppression (2). These limitations highlight an ongoing need for complementary or preventive approaches that may enhance therapeutic efficacy while reducing treatment-related burden. In this context, naturally occurring bioactive compounds with pleiotropic immunomodulatory properties have gained increasing attention. Both dietary components and environmental exposures have long been recognized as potential modulators of immune function and disease susceptibility in autoimmune conditions. Among these, phytoestrogens (plant-derived molecules structurally similar to 17β-estradiol) have emerged as particularly interesting candidates due to their ability to influence immune responses across multiple experimental systems (3). Genistein (4′,5,7-trihydroxyisoflavone), one of the most abundant isoflavones in soy and soy-derived products, is a prominent example of such compounds. In addition to its relatively weak estrogenic activity, genistein exerts a range of estrogen receptor-independent effects, including inhibition of protein tyrosine kinases and modulation of intracellular signaling pathways that are central to immune cell activation and regulation (4). These properties position genistein as a biologically relevant modulator of immune homeostasis rather than a simple dietary phytochemical. A growing body of preclinical evidence supports its immunomodulatory potential. *In vitro* and *in vivo* studies have shown that genistein can regulate both innate and adaptive immune responses, cytokine production, and immune cell differentiation. In experimental autoimmune encephalomyelitis, a widely used model of multiple sclerosis, genistein has been shown to reduce disease severity, delay clinical onset, and attenuate neuroinflammatory processes through the modulation of key inflammatory signaling pathways (5, 6). Importantly, these effects appear to be highly context-dependent, varying with dose, timing of exposure, and the hormonal status of the host - an aspect of particular relevance in light of the well-documented sex differences in autoimmune disease prevalence and course (4). In recent years, research interest in genistein has gradually shifted from mechanistic and preclinical studies toward translational and clinical exploration. Genistein-rich soy isoflavone preparations have been investigated in human studies in conditions such as metabolic syndrome and thyroid dysfunction. In a double-blind, randomized, placebo-controlled trial, genistein supplementation improved thyroid function parameters and was associated with reduced levels of thyroid-specific autoantibodies in patients with subclinical hypothyroidism, suggesting a potential modulatory effect on adaptive immune responses (7). Although preliminary observations in other autoimmune conditions are encouraging, well-designed, disease-specific clinical trials remain limited.

Against this background, this review critically summarizes current knowledge on the role of genistein in autoimmune diseases, integrating evidence from cellular studies, animal models, and clinical investigations, with a particular emphasis on its translational potential and relevance for future therapeutic strategies.

## 2 Molecular mechanisms of genistein

Genistein is a soy-derived isoflavone classified as a phytoestrogen, a group of plant compounds with estrogen-like biological activity. Interest in isoflavones emerged in the 1940s following reports of reproductive abnormalities in sheep grazing on clover rich in these compounds, which led to early recognition of their endocrine activity in mammals. In subsequent decades, experimental studies in animal models confirmed estrogenic effects of isoflavones, which were even considered for use as growth-promoting feed additives. Later research revealed a context-dependent pharmacological profile, demonstrating both estrogenic and anti-estrogenic properties depending on hormonal milieu. These findings enabled the identification and structural characterization of individual isoflavones, including genistein (8). Isoflavones, including genistein, daidzein, and glycitein, constitute a distinct subclass of phytoestrogens that modulate estrogen receptor–mediated signaling. Genistein is considered the most biologically active soy isoflavone. Structurally, phytoestrogens share similarity with 17β-estradiol, enabling interaction with both ERα and ERβ and resulting in partial modulation of estrogen-dependent signaling pathways across target tissues (9).

### 2.1 Tyrosine kinase inhibition

Genistein is a naturally occurring protein tyrosine kinase inhibitor first characterized by Akiyama et al. (1987), who demonstrated its ability to inhibit tyrosine-specific protein kinases *in vitro*, establishing a mechanistic basis for its effects on intracellular signaling pathways (10). Subsequent studies have shown that genistein is not strictly selective and may modulate multiple signaling pathways in a context-dependent manner. In immune cells, early evidence indicated that genistein influences antigen receptor-mediated signaling. In T lymphocytes, inhibition of tyrosine phosphorylation was associated with reduced activation and proliferation following CD28 or TCR stimulation, including decreased interleukin-2 production (11), suggesting suppression of key pathways involved in T cell-driven immune responses. In B cell-derived systems, genistein inhibited tyrosine phosphorylation triggered by membrane immunoglobulin cross-linking and reduced Epstein-Barr virus reactivation in Akata cells, including BZLF1 mRNA and ZEBRA protein expression (12), indicating interference with activation-linked transcriptional programs. In addition, proteomic analyses have shown that genistein broadly modulates phosphorylation networks, affecting receptor tyrosine kinases such as EGFR, PDGFR, insulin receptor, and Src-family kinases (13). These effects converge on major downstream pathways, including MAPK and PI3K/AKT signaling, which are central to immune cell activation and inflammatory responses. Overall, these findings support a model in which genistein modulates tyrosine kinase-dependent signaling at multiple levels, with relevance to immune regulation and potentially to autoimmune and inflammatory disease mechanisms.

### 2.2 Modulation of NF-κB and MAPK pathways

Genistein modulates several key intracellular signaling pathways, with particular relevance to NF-κB-mediated regulation of inflammatory responses, cellular proliferation, and stress-related signaling. *In vitro* studies in immune and synovial cell systems have shown that genistein can suppress NF-κB activation, which is associated with reduced IκBα phosphorylation and/or stabilization of IκBα, thereby limiting nuclear translocation of NF-κB subunits such as p65 and p50. This results in decreased expression of NF-κB-dependent pro-inflammatory mediators, including TNF-α, IL-1β, IL-6, and IL-8 (14, 15). In addition to NF-κB signaling, genistein has been reported to modulate mitogen-activated protein kinase (MAPK) pathways in a context-dependent manner. In LPS-stimulated macrophages, genistein attenuates phosphorylation of ERK1/2, JNK, and p38 MAP kinases, leading to reduced activation of downstream transcription factors such as AP-1 and decreased production of pro-inflammatory mediators (15). In epithelial cell models, differential regulation of MAPK signaling has been observed, including inhibition of ERK1/2 and JNK with variable effects on p38, ultimately converging on reduced AP-1 activity and an overall anti-inflammatory outcome (16). Overall, these findings suggest that genistein modulates inflammatory signaling networks through effects on both NF-κB and MAPK pathways, predominantly by influencing upstream signaling events that regulate kinase activation and transcription factor activity. This multi-level regulation likely contributes to its broad anti-inflammatory activity observed across experimental models.

### 2.3 Antioxidant effects

Genistein also exhibits notable antioxidant properties that contribute to its broader effects on cellular signaling and stress-response regulation. *In vitro* studies have shown that it reduces intracellular reactive oxygen species (ROS) accumulation and attenuates oxidative stress in a variety of cell models exposed to pro-oxidant conditions, including hydrogen peroxide (H_2_O_2_). This antioxidant action is accompanied by the enhancement of endogenous defense systems, including superoxide dismutase (SOD), catalase (CAT), and components of the glutathione-dependent antioxidant network, and is closely associated with activation of the nuclear factor erythroid 2-related factor 2 (Nrf2) pathway. Mechanistically, genistein has been reported to promote both the expression and nuclear translocation of Nrf2, leading to the transcriptional upregulation of cytoprotective phase II detoxifying and antioxidant genes, including heme oxygenase-1 (HO-1), among others, in epithelial and endothelial cell systems. Through these coordinated effects, genistein helps limit oxidative damage to cellular macromolecules while simultaneously reshaping redox-sensitive signaling pathways. Attenuation of ROS levels contributes to secondary modulation of key inflammatory cascades, including NF-κB and MAPK signaling, thereby integrating its antioxidant and immunomodulatory actions into a coherent functional network (17–19).

### 2.4 Estrogen receptor-mediated immunomodulation

Genistein, a soy-derived isoflavone structurally analogous to 17β-estradiol, exerts estrogen-like activity primarily through interaction with estrogen receptors (ERs), thereby influencing gene transcription and downstream signaling cascades (20). Owing to this structural similarity, it can bind to ERs and modulate the expression of estrogen-responsive genes across a range of cell types, acting as a weak, context-dependent modulator of estrogenic signaling rather than a full agonist or antagonist (20, 21). Within the immune system, ER signaling is an important regulator of thymic development and immune homeostasis. Experimental studies suggest that genistein can influence immune function through both ER-dependent and ER-independent mechanisms, including changes in thymic architecture and modulation of adaptive immune responses (21). In animal models, exposure to genistein has been associated with thymic involution and alterations in both humoral and cell-mediated immunity, with at least part of these effects mediated via ER-dependent pathways (21). Mechanistically, genistein may compete with endogenous estrogens, such as 17β-estradiol, for ER binding, thereby shifting the balance of receptor activation and modifying antigen-specific immune responses (22). This competitive interaction underlies its classification as a phytoestrogen with selective estrogen receptor-modulating properties rather than a classical endocrine agonist.

Beyond direct ER signaling, genistein also interfaces with broader immunoregulatory networks, including cytokine production and inflammatory signaling pathways, contributing to its overall anti-inflammatory and immunomodulatory profile (18). Importantly, its biological effects are highly context-dependent, varying according to dose, developmental stage, and cellular environment. As a result, genistein may exert either immunosuppressive or immunostimulatory effects, reflecting the pleiotropic nature of ER-associated and non-ER signaling pathways and the complexity of endocrine-immune system crosstalk (19).

At the same time, ER-mediated activity of genistein may have adverse consequences under conditions of high exposure or during sensitive developmental windows. Experimental data indicate that excessive or early-life exposure can result in thymic atrophy, impaired lymphocyte development, and reduced antigen-specific immune responses, suggesting that inappropriate modulation of ER signaling may lead to functional immunosuppression (21, 22). Furthermore, developmental exposure has been associated with long-term alterations in endocrine and reproductive function, consistent with persistent disruption of endogenous estrogen signaling pathways (23).

Collectively, these findings support the view that genistein functions as a multifunctional immunomodulatory compound that integrates estrogen receptor signaling with broader regulatory networks governing immune homeostasis, while simultaneously underscoring the critical importance of dose, timing, and biological context in determining its net physiological outcome.

### 2.5 Anti-inflammatory activity

The anti-inflammatory properties of genistein have been extensively summarized in recent review literature, which integrates data from a broad range of experimental systems and inflammatory models. Genistein is consistently described as a modulator of key inflammatory signaling pathways, including, but not limited to, cyclooxygenase-2 (COX-2)-dependent prostaglandin synthesis, a central axis in the regulation of inflammatory responses. Prostaglandin E2 (PGE2), a major downstream effector of COX-2 activity, is closely associated with classical features of inflammation such as pain, oedema, and vascular alterations (4, 24). In addition, genistein has been shown to affect higher-level regulatory networks, including transcriptional pathways such as NF-κB and MAPK, which play fundamental roles in the regulation of cytokine expression and inflammatory enzyme production. Collectively, the available evidence supports the concept that genistein functions as a multi-target modulator of inflammatory signaling rather than acting through a single defined pathway (4, 24).

## 3 Effects of genistein on innate and adaptive immunity

**Figure 1:**
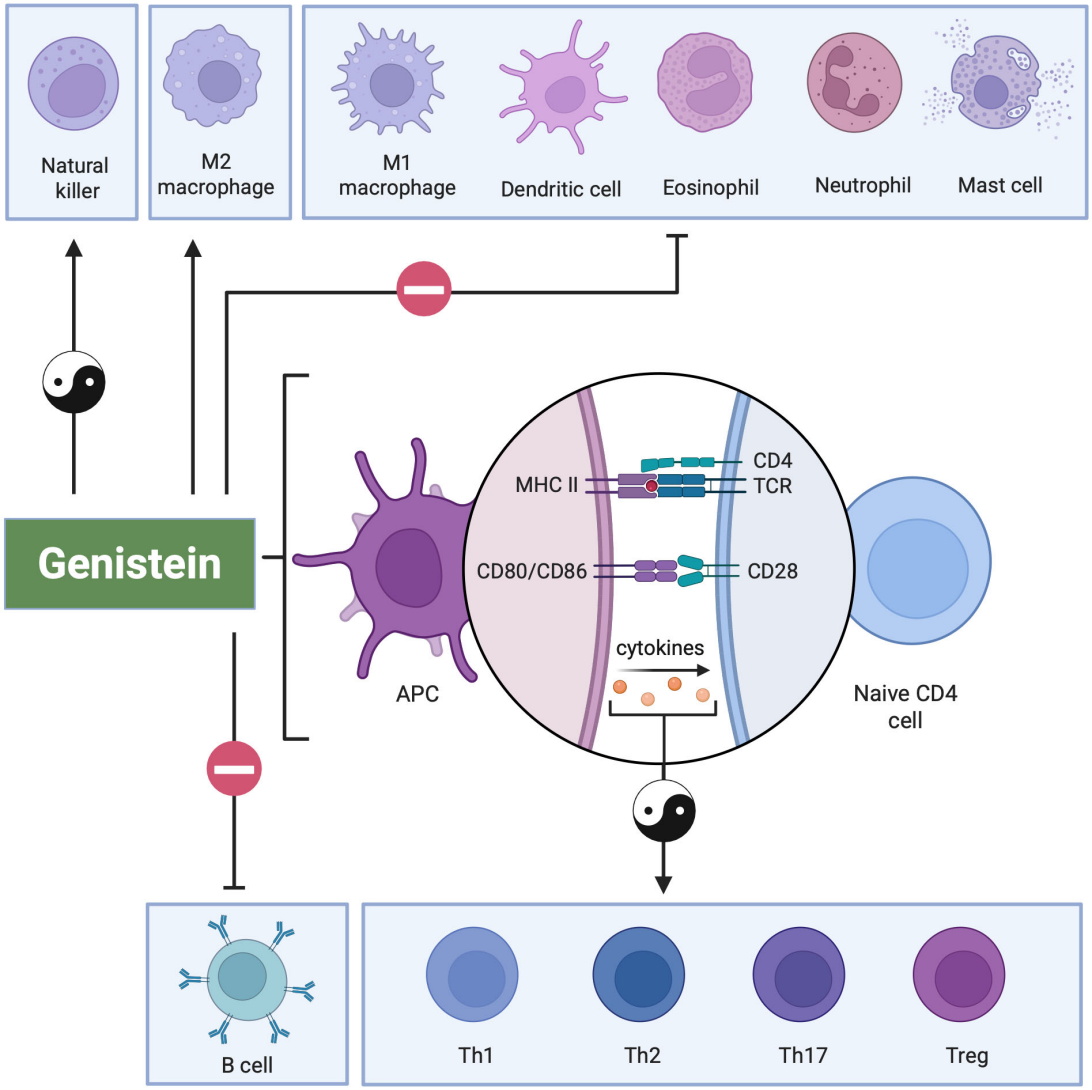
Immunomodulatory effects of genistein on innate and adaptive immune responses. Genistein exerts pleiotropic and context-dependent effects on multiple immune cell populations involved in inflammation and immune regulation. Within the innate immune compartment, genistein modulates the activity and functional polarization of natural killer (NK) cells, macrophages (M1/M2), dendritic cells, eosinophils, neutrophils, and mast cells, with either inhibitory or stimulatory effects depending on the biological context, developmental stage, activation status, dose, and experimental conditions. Genistein also influences antigen-presenting cell (APC)-mediated activation of naïve CD4+ T cells through modulation of TCR or co-stimulatory signaling (CD80/CD86-CD28), and cytokine production. These effects contribute to altered differentiation and functional balance among T helper (Th1, Th2, and Th17) and regulatory T (Treg) cell subsets, which may be either suppressed or promoted depending on the immunological milieu. In addition, genistein modulates B-cell responses and antibody-related immunity. Created with BioRender.com.

Genistein is a pleiotropic immunomodulator affecting both innate and adaptive immune responses mainly through inhibition of tyrosine kinase–dependent signaling and downstream NF-κB, MAPK, and PI3K/AKT pathways. Overall, it tends to suppress excessive inflammatory activation while modulating immune cell function in a context-dependent manner. In innate immunity, genistein reduces activation of macrophages and dendritic cells by inhibiting TLR-driven signaling, leading to decreased production of pro-inflammatory cytokines (e.g., TNF-α, IL-6, IL-1β) and reduced expression of co-stimulatory molecules. It also attenuates neutrophil degranulation and mast cell activation through modulation of MAPK- and TRAM-dependent pathways, thereby limiting release of inflammatory mediators. In contrast, NK cell activity may be enhanced under stimulatory conditions, indicating context-dependent regulation of innate cytotoxicity. In adaptive immunity, genistein suppresses early T-cell activation, reducing IL-2 production, proliferation, and signaling downstream of TCR/CD28. It also influences T-cell differentiation, shifting Th1/Th2 balance and modulating Th17 and Treg responses via transcriptional and kinase-dependent mechanisms. In B cells, it interferes with BCR signaling, antibody responses, and survival pathways, affecting both physiological and malignant B-cell functions. Collectively, genistein broadly regulates immune responses by dampening pro-inflammatory signaling while reshaping both innate and adaptive immune cell activity in a stimulus-dependent manner (10–12, 15, 19, 25–47) (**Figure 1**).

### 3.1 Innate immunity

#### 3.1.1 Macrophages

Genistein has been consistently shown to modulate macrophage inflammatory responses across multiple experimental settings, primarily by suppressing pro-inflammatory activation and promoting anti-inflammatory phenotypes. In LPS-stimulated macrophages, genistein and its derivatives reduce the production of inflammatory mediators, including nitric oxide (NO), TNF-α, IL-6, and IL-1β, as well as COX-2 expression and macrophage activation markers (CD80, CD86). These effects are associated with inhibition of TLR4-dependent NF-κB and MAPK (ERK1/2, p38, JNK) signaling pathways, indicating suppression of canonical pro-inflammatory signaling in activated macrophages (26). In RAW 264.7 macrophages, genistein also attenuates oxidative stress and inflammatory activation in a dose-dependent manner by decreasing NO and TBARS levels while enhancing intracellular antioxidant defenses, including GSH content and the activity of enzymes such as superoxide dismutase (SOD) and catalase. This is accompanied by suppression of NF-κB DNA-binding activity, further supporting inhibition of NF-κB–driven transcriptional programs in macrophage inflammation (19). Additional evidence indicates that genistein can regulate macrophage inflammatory responses through energy-sensing pathways. In LPS-treated RAW 264.7 cells, genistein reduces TNF-α and IL-6 production and suppresses NF-κB activation, while restoring AMPK phosphorylation. The anti-inflammatory effect is diminished by AMPK inhibition, suggesting that AMPK activation contributes to genistein-mediated suppression of NF-κB signaling in macrophages (15). Beyond classical inflammatory inhibition, genistein also modulates macrophage polarization. In IL-4-polarized macrophages, genistein increases expression of AhR and CYP1A1 and enhances M2-associated markers such as CD163 and arginase-1 (Arg-1), along with IL-10 expression. These effects are reversed by an AhR antagonist, indicating that genistein promotes anti-inflammatory M2 polarization via AhR-dependent signaling (27). Consistently, *in vivo* and ex vivo models of intestinal inflammation demonstrate that genistein shifts macrophage polarization from an M1 toward an M2 phenotype, reduces systemic cytokine levels, and alleviates inflammatory pathology (28).

Collectively, these findings indicate that genistein exerts multifaceted regulatory effects on macrophages, including suppression of TLR4/NF-κB-driven inflammatory activation, modulation of oxidative stress responses, and promotion of anti-inflammatory M2 polarization, thereby contributing to the resolution of inflammatory responses across different experimental models.

#### 3.1.2 Dendritic cells

Isoflavones, including genistein, have been shown to suppress dendritic cell (DC) activation in response to inflammatory stimuli such as LPS. In human monocyte-derived DCs, isoflavone treatment reduces the expression of maturation and co-stimulatory markers (CD80, CD83, CD86), thereby limiting DC activation status and functional capacity. These changes translate into impaired ability of DCs to induce effector T-cell responses, including reduced IFN-γ production in allogeneic CD4^+^ T cells, indicating suppression of Th1-polarizing potential (29). In addition, genistein reduces IL-6 production and transcription in LPS-stimulated human DCs by inhibiting NF-κB-dependent gene expression. This effect involves altered NF-κB nuclear translocation and DNA-binding activity downstream of TLR4, with relatively modest effects on upstream canonical signaling. Importantly, genistein also increases p53 protein levels, and functional studies demonstrate a regulatory cross-talk between p53 and NF-κB, where p53 negatively regulates NF-κB–driven transcription. Evidence from p53-deficient Bs further confirms that p53 is required for full suppression of IL-6 expression by genistein, highlighting a p53-NF-κB-dependent mechanism in DC inflammatory regulation (30). Collectively, these findings indicate that genistein-containing isoflavones suppress DC maturation and cytokine production, thereby attenuating DC-driven effector T-cell activation and modulating innate-adaptive immune crosstalk during inflammatory responses.

#### 3.1.3 Neutrophils

Genistein acts as a broad-spectrum tyrosine kinase inhibitor and suppresses fMLP-induced neutrophil degranulation in a compartment-specific manner. It markedly inhibits the release of primary and secondary granules, while having only a minor effect on secretory vesicle exocytosis. This is accompanied by reduced phosphorylation of key signaling intermediates, including p38 MAPK and components of upstream tyrosine kinase pathways, which are known to regulate granule mobilization in neutrophils (31) Overall, these findings indicate that genistein interferes with tyrosine kinase-dependent signaling required for full activation of neutrophil degranulation programs. In parallel, broader signaling studies indicate that genistein can differentially regulate MAPK pathways in a context-dependent manner. While p38 MAPK activity is linked to inflammatory degranulation processes, genistein has also been shown in other cellular systems to activate ERK1/2- and PI3K-dependent signaling cascades involved in cell cycle regulation and differentiation, highlighting its pleiotropic and stimulus-dependent effects on MAPK signaling networks (32). Collectively, these data suggest that genistein modulates neutrophil effector functions primarily through inhibition of tyrosine kinase-driven p38 MAPK activation, while its broader biological effects reflect context-specific regulation of MAPK-associated signaling pathways.

#### 3.1.4 NK cells

Genistein has been shown to modulate natural killer (NK) cell–related immune functions in a context-dependent and dose-dependent manner. *In vivo* studies in Sprague-Dawley rats demonstrated that dietary genistein exposure alters splenic NK cell proportions and activity in a sex- and generation-dependent manner. In parental animals, genistein increased NK cell activity, whereas in offspring it primarily affected NK cell frequencies without consistently changing cytotoxic function. These alterations occurred alongside broader changes in lymphoid organ development and T- and B-cell compartments, indicating systemic immunomodulation across both innate and adaptive immunity (33). In a complementary mouse model, genistein enhanced IL-2–stimulated NK cell cytotoxic activity at higher doses, while basal NK activity remained largely unchanged. This was accompanied by increased cytotoxic T-cell function and improved host resistance against B16F10 tumor challenge, reflected by reduced tumor nodule formation. Importantly, these effects were not due to direct tumor cytotoxicity but were associated with enhanced cell-mediated immune responses (34). Collectively, these findings suggest that genistein selectively modulates NK cell function depending on immune activation state and biological context, contributing to broader regulation of innate immune effector responses and anti-tumor immunity.

#### 3.1.5 Mast cells

Genistein has been shown to suppress mast cell inflammatory activation induced by PMA/A23187 stimulation. In activated mast cells, it significantly reduces IL-6 and IL-1β mRNA expression, as well as IL-6 production, indicating inhibition of key pro-inflammatory cytokine output. Mechanistically, this effect is associated with selective inhibition of ERK1/2 phosphorylation, while p38 MAPK activation remains largely unaffected. Overall, these findings suggest that genistein attenuates mast cell-driven inflammatory responses primarily through modulation of ERK-dependent signaling pathways, leading to reduced production of pro-inflammatory mediators (35). In addition, genistein has been identified as a potential inhibitor of mast cell-mediated anaphylactoid responses driven by MRGPRX2 activation. In experimental models, it suppresses MRGPRX2-dependent mast cell activation, resulting in reduced vascular permeability and decreased edema formation *in vivo*. These effects translate into attenuation of anaphylactoid shock–like responses, indicating interference with non–IgE-mediated mast cell activation pathways (36). Furthermore, in mast cell-driven inflammatory disease models, including atopic dermatitis, genistein reduces disease severity as evidenced by decreased tissue inflammation, scratching behavior, and systemic allergic markers such as IgE and IL-4. These effects are accompanied by inhibition of mast cell degranulation and reduced release of histamine, β-hexosaminidase, tryptase, MCP-1, and TNF-α. Mechanistically, genistein targets TRAM within Toll-like receptor signaling, directly binding to this adaptor protein and inhibiting downstream PLCγ1-IKKβ-NF-κB signaling, thereby suppressing mast cell activation and inflammatory mediator release (37). Collectively, these findings demonstrate that genistein modulates mast cell function through multiple mechanisms, including inhibition of ERK-dependent cytokine production, blockade of MRGPRX2-mediated activation, and suppression of TRAM-dependent TLR signaling, ultimately attenuating mast cell–driven inflammatory responses across different pathological contexts.

### 3.2 Adaptive immunity

#### 3.2.1 T cells

Genistein acts as a protein tyrosine kinase (PTK) inhibitor and was early identified as a classical inhibitor of tyrosine-specific protein kinases, including receptor and non-receptor kinases such as the EGF receptor and Src family kinases. In biochemical assays, it inhibits kinase activity in an ATP-competitive manner while showing minimal effects on serine/threonine kinases, supporting preferential targeting of tyrosine phosphorylation–dependent signaling pathways (10). Subsequent studies in T cells showed that although genistein can inhibit T-cell receptor (TCR)-dependent signaling, its effects are partial and not fully specific for PTK inhibition in this context. In Jurkat T cells, it reduces TCR-mediated phospholipase C (PLC) activation and downstream signaling but also affects alternative signaling pathways and exerts broader cellular effects, including cytotoxicity and inhibition of protein synthesis. These observations suggest that genistein modulates T-cell signaling through additional mechanisms beyond selective PTK inhibition (38). At the level of T-cell activation, genistein inhibits CD3- and CD28-dependent signaling pathways, reducing IL-2 production, IL-2 receptor (IL-2R) expression, CD2 upregulation, and proliferation of peripheral blood mononuclear cells (PBMCs). These effects are consistent with interference in early tyrosine phosphorylation–dependent signaling events required for full T-cell activation. Importantly, genistein suppresses pathways that are partially resistant to calcineurin inhibition, indicating involvement of CsA-independent signaling routes (11). Functional studies in normal and malignant lymphocytes further demonstrated that genistein strongly affects lymphocyte proliferation and cell cycle progression. In mitogen-stimulated normal lymphocytes, it inhibits the G0→G1 transition, whereas in leukemic cell lines (e.g., HL-60 and MOLT-4) it induces S- or G2-phase arrest and, at higher concentrations, cytotoxic and apoptotic effects. These data indicate that genistein targets kinase-dependent regulation of cell cycle progression, linking tyrosine kinase signaling with lymphocyte activation and proliferation control (39). Beyond early activation signals, genistein modulates T-cell differentiation and effector polarization. In allergic airway inflammation models, it shifts the Th1/Th2 balance by decreasing Th2-associated cytokines (IL-4, IL-5) and increasing Th1-associated IFN-γ, accompanied by regulation of transcription factors such as downregulation of GATA-3 and STAT-6 and upregulation of T-bet. This indicates an effect on transcriptional programming of T-cell responses (40). In regulatory T-cell (Treg) biology, genistein reduces Treg differentiation and suppressive function in tumor microenvironments, enhancing CD8^+^ T-cell responses and improving responses to anti-PD-1 therapy. Mechanistically, this is linked to inhibition of PI3K/AKT signaling, a pathway essential for Treg stability and function (41). Conversely, genistein can also influence Treg-associated immune homeostasis in non-malignant contexts. In aged gut models, it improves intestinal integrity and reduces inflammation partly through Treg-derived IL-10-dependent mechanisms, linking adaptive immune regulation with tissue homeostasis (42). In Th17-related pathways, genistein has been reported to modulate RORα/γ nuclear receptor activity and enhance IL-17A transcription in experimental systems. This involves increased interaction between ROR receptors and co-activators, suggesting context-dependent effects on Th17 differentiation programs (43). Finally, in autoimmune neuroinflammation, genistein and its derivatives attenuate experimental autoimmune encephalomyelitis (EAE), reducing IL-17–producing CD4^+^ T cells and increasing Foxp3^+^ regulatory T cells, along with enhanced IL-10 and CTLA-4 expression and reduced pro-inflammatory cytokines such as IL-6 and IFN-γ. These findings indicate a shift toward a more regulatory immune phenotype in T-cell–mediated autoimmune disease (31).

Genistein broadly modulates T cell function by inhibiting early activation signals, mainly through interference with tyrosine kinase-dependent pathways downstream of TCR/CD28 stimulation. This leads to reduced IL-2 production, IL-2R expression, and T cell proliferation. Beyond activation, it influences T cell differentiation, shifting Th1/Th2 balance, affecting Th17-related pathways, and modulating regulatory T cell (Treg) responses in a context-dependent manner. Overall, genistein acts as a pleiotropic immunomodulator affecting T cell activation, polarization, and immune regulation depending on the biological setting.

#### 3.2.2 B cells

Early evidence indicated that genistein interferes with B-cell receptor (BCR)–associated signaling pathways by modulating tyrosine phosphorylation–dependent processes required for antigen receptor trafficking. In human pre-B cells (Nalm-6), cross-linking of surface immunoglobulin triggers rapid internalization of the pre-B cell receptor (pre-BCR), a process dependent on reversible tyrosine phosphorylation. Pharmacological studies showed that inhibition of tyrosine kinase activity by genistein dose-dependently blocks pre-BCR endocytosis without affecting unrelated trafficking pathways, such as transferrin receptor internalization. These findings indicate that balanced tyrosine phosphorylation and dephosphorylation are essential for antigen receptor dynamics during early B-cell development, and that genistein disrupts this regulatory system (44). Subsequent studies in EBV-infected B cells demonstrated that activation of phosphotyrosine kinases is required for viral reactivation following BCR cross-linking. In Akata cells, anti-IgG stimulation induces rapid tyrosine phosphorylation and triggers the lytic EBV cycle, including expression of immediate-early viral genes. Genistein at non-cytotoxic concentrations inhibits these phosphorylation events and blocks EBV reactivation, as shown by reduced expression of BZLF1 mRNA and its protein product ZEBRA. The effect is reversible and depends on early exposure during signaling initiation, indicating that genistein acts at an early stage of BCR-triggered signaling required for viral gene induction (12). Functional immunomodulatory effects of genistein on humoral immunity were further demonstrated *in vivo* in models of allergen sensitization. Perinatal exposure to genistein alters long-term antibody responses, particularly IgE production following exposure to respiratory allergens such as trimellitic anhydride (TMA). In adult mice, developmental exposure leads to sex- and timing-dependent modulation of IgE levels, associated with altered regulatory T cell frequencies, changes in B-cell activation markers (e.g., CD86), increased cytokine production (IL-2, IL-4), and enhanced innate immune activity. These data indicate that genistein exposure during immune system development induces persistent changes in B-cell–mediated humoral responses and allergic sensitization (45). Similarly, exposure to the soy isoflavone metabolite equol enhances antigen-specific humoral immunity *in vivo*. In ovalbumin-immunized mice, equol increases OVA-specific IgE production without major changes in IFN-γ or IL-4, but with a selective increase in IL-13, suggesting skewing toward Th2-associated antibody responses. This effect persists in ovariectomized animals, indicating independence from endogenous estrogen signaling and supporting a direct immunomodulatory action on B-cell–associated immune pathways (46). In malignant B cells, genistein exerts antiproliferative and pro-apoptotic effects by altering cytokine signaling and cell cycle regulation. In chronic B-cell malignancies with autocrine IL-10 production, genistein reduces IL-10 secretion and increases IFN-γ expression, leading to inhibition of proliferation and G2/M cell cycle arrest. Mechanistically, this is associated with downregulation of key cell cycle regulators (cdc25C, CDK1) and anti-apoptotic proteins (survivin, IAN5). The growth-inhibitory effect is reversible and can be counteracted by exogenous IL-10, indicating a central role of cytokine modulation in genistein activity (47).

Genistein modulates B-cell biology at multiple levels, including antigen receptor signaling, antibody (IgE) responses, viral reactivation in EBV-infected B cells, and malignant B-cell proliferation. Overall, its effects converge on tyrosine kinase–dependent signaling and cytokine-regulated control of B-cell activation and survival.

## 4 Preclinical models

The PubMed-based literature identified using the search strategy “autoimmune diseases AND genistein” indicates that genistein has been investigated across a broad range of experimental autoimmune and immune-mediated disease models. These include systemic lupus erythematosus (SLE), rheumatoid arthritis, experimental autoimmune encephalomyelitis, type 1 diabetes, Sjögren’s syndrome, autoimmune thyroid disease, autoimmune ovarian disease, and autoimmune blistering skin disorders (**Table 1**). Across these diverse systems, the available evidence consistently points to an ability of genistein to modulate immune responses, inflammatory signaling pathways, and tissue-specific pathological processes. However, it is important to note that the depth of mechanistic insight varies considerably between disease models, with some contexts being supported by relatively detailed molecular data, while others remain at a more descriptive or exploratory stage.

**Table 1:** Preclinical and clinical evidence on the effects of genistein in autoimmune diseases.

Study type	Disease / model	Key findings	Mechanisms	Ref.
In vivo (MRL/lpr, pristane-induced mice)	SLE	Reduced proteinuria, improved renal histopathology, decreased autoantibodies and IL-6	ERβ activation; inhibition of STAT3 and NF-κB; suppression of M1 macrophage polarization	(48)
In vivo (CIA model)	RA	Reduced disease severity	Modulation of Th1/Th2 balance; decreased pro-inflammatory cytokines	(51)
In vitro (MH7A cells)	RA	Anti-inflammatory effects in synoviocytes	Inhibition of ROS/Akt/NF-κB; activation of AMPK	(14)
In vivo (CIA model)	RA	Reduced joint inflammation, preserved cartilage integrity	↓ TNF-α, ↓IL-1β, ↓IL-6, ↓MMP-9; inhibition of exosome secretion; suppression of Wnt/β-catenin	(52)
In vivo (EAE model)	MS/EAE	Reduced disease severity, decreased rolling and adhering of leukocytes in CNS	Modulation of pro- and anti-inflammatory cytokines	(5)
In vivo (EAE, TDG derivative)	MS/EAE	Improved outcomes, reduced inflammatory changes and demyelination	↑ Treg, ↑ IL-10; ↓ IL-17, ↓ IFN-γ, ↓ IL-6	(54)
In vivo (EAE)	MS/EAE	Reduced demyelination	↓ IFN-γ, ↓TNF-α, ↓IL-12, ↑ IL-10	(55)
In vivo (EAE)	MS/EAE	Modulation of innate immunity	↑ TLR3, ↑ TLR9; ↑ IFN-β	(6)
In vivo (NOD mice)	T1D	Delayed onset, reduced incidence (adults); sex-dependent effects	Microbiota modulation; cytokine regulation	(58, 59)
In vivo (STZ model)	T1D complications	Improved wound healing, angiogenesis	↓ oxidative stress; inhibition of FoxO1/iNOS	(60)
In vivo + in vitro	Sjögren’s syndrome	Improved gland function	ERα → Xist ↑ → ACSL4 ↓ → ferroptosis inhibition	(62)
In vivo (mouse model)	Autoimmune ovarian disease	Dose-dependent (protective at high doses)	Estrogen-like effects; hormonal modulation	(63)
In vitro	Pemphigus vulgaris	Inhibition of Dsg3 internalization	Blockade of endocytosis	(65)
In vitro	Pemphigus vulgaris	Reduced signaling	Inhibition of tyrosine kinase, p38 MAPK	(66)
In vivo (mouse model)	Pemphigus vulgaris	Prevention of epidermal damage	↓ iNOS/nNOS; inhibition of NF-κB	(67)
In vivo (mouse model)	IMQ-induced psoriasis	Improved clinical and histological parameters	↓ IL-1β, ↓ IL-6, ↓ TNF-α, ↓ CCL2, ↓ IL17, ↓ IL-23; inhibition of STAT3 and NF-κB activity	(69)
Clinical RCT	Hashimoto’s thyroiditis	Increases total and free thyroxine (T4 and fT4), reduction TSH and autoantibodies against TPO and Tg	↓ IL-2, ↑ IFN-γ	(7)
Clinical RCT	Psoriasis	A borderline effect was noted for Physician’s Global Assessment at day 56 (p = 0.0506)	Inhibition of MAPK, NF-κB, and PI3K activation	(79)

### 4.1 Systemic lupus erythematosus

Systemic lupus erythematosus (SLE) is a chronic, systemic autoimmune disorder characterized by a breakdown of immune tolerance and the subsequent production of autoantibodies, leading to widespread inflammation and multi-organ tissue damage. In experimental models of SLE, genistein has been shown to attenuate disease activity and mitigate renal injury. In MRL/lpr and pristane-induced lupus mouse models, genistein administration reduced proteinuria, improved renal histopathological features, and decreased circulating levels of autoantibodies as well as interleukin-6 (IL-6). These protective effects were accompanied by suppression of macrophage M1 polarization and downregulation of key inflammatory signaling pathways, including STAT3 and NF-κB. Mechanistically, the immunomodulatory actions of genistein in these models appear to involve, at least in part, activation of estrogen receptor β (ERβ), as pharmacological inhibition of ERβ partially reversed its anti-inflammatory effects (48).

### 4.2 Rheumatoid arthritis

Rheumatoid arthritis (RA) is a chronic systemic autoimmune disease characterized by synovial inflammation, hyperplasia of fibroblast-like synoviocytes, progressive destruction of cartilage and bone, and the presence of autoantibodies such as rheumatoid factor and anti-citrullinated protein antibodies (ACPA) (49, 50). *In vivo*, genistein has been shown to attenuate disease severity in the collagen-induced arthritis (CIA) model, accompanied by modulation of immune responses, including a shift in the Th1/Th2 balance and a reduction in pro-inflammatory cytokine production (51). Complementary *in vitro* studies further support its anti-inflammatory activity in RA-relevant cellular systems, demonstrating that genistein modulates ROS/Akt/NF-κB signaling and AMPK pathways in MH7A synoviocytes, thereby reinforcing its potential relevance for pharmacological exploration in RA (14). Additional evidence extends these observations, showing that genistein can alleviate arthritis severity in CIA mice by reducing joint inflammation and preserving cartilage integrity. These effects are associated with decreased expression of key pro-inflammatory mediators, including TNF-α, IL-1β, IL-6, and MMP-9 in fibroblast-like synoviocytes. Mechanistically, this study highlights inhibition of exosome secretion via the Rab27/nSMase2/Mfge8 axis, along with suppression of Wnt/β-catenin signaling and miR-221-3p expression. Collectively, these findings support a multi-level regulatory role of genistein in the pathophysiology of RA (52).

### 4.3 Neuroinflammatory autoimmune disease: EAE model

Multiple sclerosis (MS) is a chronic, inflammatory demyelinating disease of the central nervous system, driven by immune-mediated damage involving autoreactive T and B lymphocytes, macrophage infiltration, and subsequent neurodegeneration (30). Experimental autoimmune encephalomyelitis (EAE) is a widely used animal model of MS and is characterized by immune-driven inflammatory demyelination within the central nervous system, accompanied by progressive neurological impairment (53). In a murine MOG_35_-_35_-induced EAE model, genistein treatment significantly reduced clinical disease severity and modulated inflammatory responses. Treated animals exhibited a pronounced reduction in pro-inflammatory cytokine production, along with a shift in immune activity toward a less inflammatory phenotype. Intravital microscopy further demonstrated decreased leukocyte rolling and adhesion within the central nervous system compared with untreated controls, indicating reduced immune cell trafficking and infiltration into inflamed neural tissue. Collectively, these findings suggest that genistein attenuates key neuroinflammatory processes in experimental models of MS (5). To enhance biological activity, more lipophilic derivatives of genistein have also been evaluated. 7-O-tetradecanoyl-genistein (TDG), a lipophilic ester derivative, improved clinical outcomes in EAE when administered after disease induction. This was associated with immunomodulation within the central nervous system, including reduced IL-17-producing cells and increased Foxp3^+^CD4^+^ regulatory T cells. In addition, elevated IL-10 production and CTLA-4 expression were observed, along with decreased IFN-γ and IL-6 levels. Histological analysis confirmed reduced inflammatory changes and demyelination in brain tissue, indicating a stronger immunoregulatory profile compared with the parent compound (54). Further studies have shown that the timing of genistein administration is critical for its efficacy. In the same EAE model, genistein administered after disease establishment did not reverse clinical symptoms, whereas treatment initiated at disease onset improved neurological outcomes and reduced demyelination. These effects were accompanied by modulation of cytokine profiles, including decreased IFN-γ, IL-12, and TNF-α, together with increased IL-10, suggesting that genistein is more effective in early phases of disease progression (55). In addition, early-stage genistein treatment has been linked to modulation of innate immune signaling pathways. This includes increased expression of Toll-like receptors TLR3 and TLR9 as well as elevated IFN-β levels, suggesting that genistein may also influence early neuroinflammatory signaling and innate immune activation in EAE (6).

Collectively, these findings indicate that genistein and its derivatives exert multi-level immunoregulatory effects in experimental autoimmune neuroinflammation, influencing both innate and adaptive immune responses and attenuating disease severity depending on timing and chemical form.

### 4.4 Type 1 diabetes

Type 1 diabetes mellitus (T1D) is an autoimmune disorder characterized by immune-mediated destruction of pancreatic β-cells, leading to absolute insulin deficiency and subsequent metabolic dysregulation (56). Experimental studies suggest that genistein may influence both the development and progression of T1D through immunomodulatory mechanisms, as well as metabolic effects that appear to be partially linked to alterations in gut microbiota composition. In a perinatal exposure model using non-obese diabetic (NOD) mice, genistein administered at 20 mg/kg from embryonic day 7 to postnatal day 21 exerted sex-dependent effects on disease susceptibility. Female offspring displayed an increased incidence of T1D, accompanied by reduced levels of IL-10, IgG2a, and IgM, as well as a shift toward a more pro-inflammatory profile in splenic immune cell populations. In male offspring, a reduction in total T cell and CD4+ T cell populations was observed. These immunological changes were associated with alterations in gut microbiota composition and intestinal α-defensin expression, suggesting a role for microbiota–immune system interactions, particularly in females (57). In contrast, studies in adult NOD mice have reported predominantly protective effects of genistein on T1D development. Daily oral administration delayed disease onset and reduced diabetes incidence in female mice maintained on a soy- and alfalfa-free diet. Higher doses (6 and 20 mg/kg) significantly decreased both total and severe diabetes incidence, whereas the lowest dose (2 mg/kg) produced only transient early protection. Histopathological analyses further confirmed preservation of pancreatic islet integrity and reduced autoimmune destruction. Notably, these protective effects were not observed in mice consuming a phytoestrogen-containing diet, indicating that background dietary phytoestrogens may modulate genistein’s efficacy (58). Additional work in adult NOD mice has also demonstrated sex-dependent effects of genistein on glucose metabolism, immune responses, and gut microbiota composition. In male mice, genistein improved glucose tolerance, reduced blood glucose levels, and induced a broadly anti-inflammatory profile characterized by decreased IgG2b, cytokines, and chemokines, alongside shifts in microbial diversity. In females, genistein delayed T1D onset; however, immune and microbiome signatures suggested a more complex, and in some contexts pro-inflammatory, immunological response despite the delayed disease progression (59). Beyond its effects on autoimmune pathways, genistein may also ameliorate diabetes-associated complications. In streptozotocin (STZ)-induced diabetic mice, genistein accelerated wound healing and enhanced angiogenesis. These effects were associated with reduced oxidative stress, inhibition of iNOS activity and FoxO1 signaling, as well as improved tissue perfusion and endothelial function (60).

### 4.5 Sjögren’s syndrome

Sjögren’s syndrome (SS) is a chronic systemic autoimmune disorder primarily characterized by sicca symptoms, including dry eyes and dry mouth, with potential multi-organ involvement (61). Experimental studies indicate that genistein may alleviate SS-related pathology through modulation of estrogen receptor α (ERα)-dependent regulation of ferroptosis in salivary gland epithelial cells (SGECs). *In vivo* studies using NOD/LtJ mice have shown that SS is associated with a ferroptosis-like phenotype in salivary glands, characterized by decreased Xist expression and increased ACSL4 expression. In this context, genistein treatment significantly improved disease-related manifestations, including reduced xerostomia and partial restoration of salivary gland function. Mechanistically, genistein was found to upregulate Xist expression via ERα signaling while concurrently downregulating ACSL4, thereby inhibiting ferroptotic processes in salivary gland tissue. *In vitro*, IFN-γ-stimulated SGECs displayed both inflammatory activation and ferroptosis, accompanied by dysregulation of XIST and ACSL4 expression. Genistein bound to ERα and promoted XIST expression, which in turn suppressed ACSL4 and contributed to the restoration of epithelial markers such as aquaporin 5. These changes were associated with reduced inflammatory signaling and reversal of ferroptosis-associated cell death. Further molecular analyses supported a direct interaction between ERα and the XIST promoter as a critical regulatory step in this pathway. Collectively, these findings suggest that genistein mitigates SS pathology by inhibiting ferroptosis through an ERα-Xist-ACSL4 regulatory axis, identifying both a potential therapeutic mechanism and Xist as a candidate molecular target in Sjögren’s syndrome (62).

### 4.6 Autoimmune ovarian disease

Autoimmune ovarian disease (autoimmune oophoritis) is a rare autoimmune disorder characterized by immune-mediated inflammation of the ovaries, leading to follicular destruction, fibrosis, and progressive ovarian atrophy. In a mouse model of experimental autoimmune ovarian disease induced by immunization with a zona pellucida–derived peptide in BALB/c females, genistein demonstrated clear dose-dependent, estrogen-like effects on disease outcome. Higher doses (25 and 45 mg/kg) were associated with improved ovarian integrity, including a more preserved histological architecture, increased proportions of developing and mature follicles, modulation of circulating sex hormone levels, and enhanced proliferative activity within ovarian tissue. These changes were accompanied by a reduction in the severity of autoimmune oophoritis. In contrast, administration of a lower dose (5 mg/kg) was associated with exacerbation of ovarian pathology and more pronounced disease manifestations. Collectively, these findings indicate a bidirectional, dose-dependent action of genistein in this model, where higher exposure levels appear to exert a protective effect against autoimmune-mediated ovarian damage, while lower doses may be insufficient to confer such benefit and may even worsen disease features (63).

### 4.7 Autoimmune blistering diseases

Pemphigus comprises a group of rare but severe autoimmune blistering disorders driven by pathogenic autoantibodies primarily targeting the desmosomal adhesion molecules desmoglein-1 (Dsg1) and desmoglein-3 (Dsg3), which are expressed in the skin (epidermis) and mucous membranes. Binding of these autoantibodies disrupts desmosomal adhesion between keratinocytes, resulting in loss of intercellular cohesion (acantholysis) and subsequent intraepidermal blister formation (64). In pemphigus vulgaris (PV) models, genistein has been shown to interfere with the pathogenic consequences of anti-Dsg3 autoantibodies. In particular, it inhibits PV IgG-induced internalization of Dsg3 in keratinocytes, a key step in the loss of cell–cell adhesion and blister development. Mechanistically, this effect is linked to inhibition of a cholesterol-sensitive, clathrin- and dynamin-independent endocytic pathway. By preventing Dsg3 endocytosis, genistein helps preserve desmosomal integrity and maintains keratinocyte cohesion, suggesting a direct stabilizing effect on epithelial junctions in PV (65). Extending these trafficking-related observations, further studies have demonstrated that genistein also modulates upstream signaling events triggered by polyclonal PV IgG. In this setting, it inhibits tyrosine kinase-dependent activation of downstream pathways, including p38 MAPK, which are required for Dsg3 clustering and internalization. As a result, inhibition of these signaling cascades prevents Dsg3 endocytosis and supports the maintenance of keratinocyte adhesion. Notably, this signaling-dependent mechanism appears to be particularly relevant in the context of polyclonal antibody responses, whereas monoclonal anti-Dsg3 antibodies can disrupt adhesion independently of tyrosine kinase- and p38 MAPK-mediated pathways, and are therefore less susceptible to genistein-mediated inhibition (66). Consistent with these mechanistic data, *in vivo* studies further support a broader protective role of genistein in PV. In murine models injected with PV-IgG, genistein pretreatment prevents the development of both clinical and histological features of acantholysis. This protective effect is associated with suppression of multiple inflammatory and redox-sensitive pathways, including reduced expression of neuronal and inducible nitric oxide synthases (nNOS and iNOS), decreased nitrotyrosine formation, and inhibition of NF-κB nuclear translocation. Collectively, these findings indicate that genistein interferes with several interconnected pathogenic mechanisms in PV, encompassing both tyrosine kinase-dependent signaling and downstream inflammatory responses, ultimately limiting antibody-induced epidermal injury (67).

To date, the immunoregulatory effects of genistein have not been investigated in subepidermal autoimmune blistering skin diseases. Accordingly, we evaluated its effects in pemphigoid disease. In a preliminary study conducted by our group, genistein reduced ROS production by activated neutrophils *in vitro* and was associated with a marked reduction in disease severity *in vivo* in the experimental model of epidermolysis bullosa acquisita. Although these findings remain unpublished, they suggest that genistein may act not only through keratinocyte-related mechanisms described in pemphigus but also by modulating key effector pathways responsible for subepidermal blister formation.

### 4.8 Psoriasis

Psoriasis has been included in the spectrum of autoimmune diseases by some authors, although this classification remains debated; in this context, it is characterized by dysregulated keratinocyte proliferation and sustained immune activation leading to persistent cutaneous inflammation (68). In an imiquimod-induced murine model of psoriasis-like skin inflammation, genistein significantly improved clinical and histopathological parameters, including reduced erythema, scaling, and epidermal hyperplasia, accompanied by a clear decrease in epidermal thickness. These changes were associated with broad suppression of the inflammatory milieu, reflected by reduced expression of key cytokines and chemokines such as IL-1β, IL-6, TNF-α, CCL2, IL-17, and IL-23 in lesional skin. Overall, the data indicate that genistein attenuates psoriasis-like inflammation through coordinated inhibition of STAT3- and NF-κB-dependent signaling pathways, leading to reduced keratinocyte activation and suppression of pro-inflammatory cytokine networks in the skin (69).

## 5 Clinical evidence

PubMed searches restricted to randomized controlled trials and clinical intervention studies identify several hundred publications evaluating genistein supplementation in humans across a wide range of physiological and disease-related contexts. However, these studies are highly heterogeneous with respect to study populations, dosing regimens, intervention duration, and outcome measures, which limits direct comparability and reduces the overall strength of generalizable conclusions. Most clinical trials have focused on non-autoimmune conditions, particularly metabolic disorders such as insulin resistance, dyslipidemia, and broader cardiometabolic risk profiles. Taken together, these studies suggest modest but consistent effects on inflammatory markers, oxidative stress parameters, and selected metabolic endpoints, supporting a potential immunometabolic role for genistein in humans. In contrast, evidence in autoimmune diseases remains extremely limited. To date, only a single randomized controlled trial has specifically evaluated genistein supplementation in Hashimoto’s thyroiditis, demonstrating a significant beneficial effect.

### 5.1 Hashimoto’s thyroiditis

Hashimoto’s thyroiditis (HT) is a common autoimmune disorder, also referred to as chronic lymphocytic thyroiditis, and represents one of the most prevalent autoimmune diseases worldwide. It is characterized by dysregulation of both cellular and humoral immunity, including infiltration of the thyroid gland by T and B lymphocytes and the production of autoantibodies against thyroid peroxidase (TPO) and thyroglobulin (Tg) (70). Progressive immune-mediated destruction of thyroid follicular cells ultimately leads to thyroid dysfunction and is a major cause of hypothyroidism. A central feature of HT pathogenesis is excessive activation of T helper cells, particularly a Th1-skewed immune response. In a double-blind, randomized, placebo-controlled clinical trial, 218 female patients with HT were randomized to receive either genistein or placebo. Genistein was administered as a purified soy extract at a dose of 600 mg/day for one month. Following the intervention, patients receiving genistein exhibited significant increases in serum total and free thyroxine (T4 and fT4), along with reductions in thyroid-stimulating hormone (TSH) and in autoantibodies against TPO and Tg compared with the placebo group. In addition, modulation of Th1-associated cytokines was observed, including changes in interferon-γ (IFN-γ) and interleukin-2 (IL-2), suggesting an effect on Th1-driven immune responses. Specifically, IL-2 levels were significantly reduced, while IFN-γ levels were increased. Overall, genistein was well tolerated and associated with both endocrine and immunomodulatory effects in patients with HT, supporting its potential role in modulating autoimmune thyroid dysfunction (7).

### 5.2 Metabolic and inflammatory conditions

Although not classified as autoimmune diseases per se, a growing number of randomized controlled trials has evaluated genistein supplementation in metabolic and inflammatory conditions characterized by chronic low-grade inflammation and immune-metabolic dysregulation. These disorders share overlapping mechanistic features with autoimmune diseases, including activation of NF-κB-dependent signaling pathways, oxidative stress, and cytokine imbalance, suggesting potential relevance for the broader immunomodulatory profile of genistein. In this context, clinical studies have been conducted in non-alcoholic fatty liver disease (NAFLD), type 2 diabetes mellitus (T2DM), metabolic syndrome, asthma, and chronic kidney disease. Although these conditions differ fundamentally in etiology from classical autoimmune disorders, they represent clinically relevant models of persistent systemic inflammation and immune activation. As such, they may provide indirect translational insight into immunometabolic mechanisms that could also be relevant to autoimmune disease pathophysiology.

#### 5.2.1 Non−alcoholic fatty liver disease

A randomized, double-blind, placebo-controlled trial investigated the effects of genistein supplementation in patients with non-alcoholic fatty liver disease (NAFLD), a hepatic manifestation of metabolic syndrome. In this study, 82 participants were randomly assigned to receive either 250 mg of genistein or placebo daily for 8 weeks, in combination with standard recommendations for dietary modification and physical activity. At the end of the intervention, the genistein-treated group demonstrated significantly reduced serum insulin levels and improved insulin sensitivity, as reflected by a decrease in HOMA-IR, compared with the placebo group. In parallel, significant reductions were observed in biomarkers of oxidative stress and inflammation, including malondialdehyde (MDA), TNF-α, and IL-6. Additional improvements were noted in selected anthropometric and metabolic parameters, such as waist-to-hip ratio, body fat percentage, and triglyceride concentrations. In contrast, no significant between-group differences were detected for body mass index, fasting blood glucose, or hepatic transaminases (ALT and AST). Overall, these findings suggest that short-term genistein supplementation may exert beneficial effects on insulin resistance and selected inflammatory and metabolic parameters in patients with NAFLD, although confirmation in larger, longer-term clinical trials is warranted (71).

#### 5.2.2 Type 2 diabetes mellitus

A randomized, double-blind, placebo-controlled clinical trial evaluated the effects of genistein supplementation in postmenopausal women with type 2 diabetes mellitus (T2DM). The study included 54 participants who were assigned to receive either genistein (108 mg/day) or placebo for 12 weeks. Anthropometric, biochemical, and lifestyle-related parameters were assessed at baseline and after the intervention. At the end of the study, genistein supplementation was associated with significant reductions in fasting blood glucose, glycated hemoglobin (HbA1c), triglycerides, and malondialdehyde (MDA), together with an increase in total antioxidant capacity (TAC), compared with the placebo group. In addition, improvements in high-density lipoprotein cholesterol and insulin sensitivity, as assessed by QUICKI, were observed within the genistein-treated group. No significant between-group differences were detected for anthropometric measures or several other metabolic parameters. Overall, these findings suggest that genistein may contribute to improved glycemic control, lipid profile, and oxidative stress parameters in postmenopausal women with T2DM, although larger and longer-duration clinical trials are required to confirm and extend these observations (72).

Collectively, these findings are consistent with a recent systematic review summarizing both preclinical and clinical evidence on the antidiabetic effects of genistein. Preclinical studies indicate reductions in blood glucose and triglyceride levels, improved insulin sensitivity, and delayed onset of both type 1 and type 2 diabetes. Clinical studies, while more modest in effect size, have reported improvements in insulin sensitivity and lipid profiles, as well as potential delays in the progression of type 2 diabetes. Overall, the available evidence suggests that genistein modulates both metabolic and immune pathways and may contribute to the attenuation of selected diabetes-related complications (73).

#### 5.2.3 Metabolic syndrome

A randomized, double-blind, placebo-controlled trial conducted in postmenopausal women with metabolic syndrome assessed the long-term effects of genistein supplementation on metabolic and cardiovascular risk markers. The study enrolled 120 participants who received either genistein (54 mg/day) or placebo for 12 months, following an initial stabilization period. After one year of intervention, genistein supplementation was associated with significant reductions in fasting glucose, fasting insulin, and insulin resistance, as measured by HOMA-IR, whereas no comparable changes were observed in the placebo group. Beneficial modifications in lipid profile were also reported, including decreases in total cholesterol, low-density lipoprotein cholesterol (LDL-C), and triglycerides, alongside an increase in high-density lipoprotein cholesterol (HDL-C). Furthermore, genistein influenced several adipokines and cardiovascular risk-related biomarkers, with increased adiponectin levels and decreased visfatin and homocysteine concentrations. Reductions in both systolic and diastolic blood pressure were also observed over the course of the intervention. The supplementation was well tolerated, with no significant differences in adverse events between the genistein and placebo groups. Collectively, these findings suggest that long-term genistein supplementation may improve multiple surrogate markers associated with cardiometabolic and diabetes risk in postmenopausal women with metabolic syndrome (74).

#### 5.2.4 Asthma

A translational study investigated the effects of genistein and soy isoflavone supplementation on eosinophilic inflammation in patients with asthma. The mechanistic *in vitro* component demonstrated that genistein inhibited eosinophil leukotriene C_4_ synthesis and modulated key signaling pathways involved in inflammatory mediator production, including suppression of p38 MAP kinase activation and 5-lipoxygenase translocation. In the short-term clinical component, patients receiving soy isoflavone supplementation (100 mg/day for 4 weeks) exhibited reductions in ex vivo eosinophil leukotriene production, as well as decreased fractional exhaled nitric oxide (FeNO), a surrogate marker of airway inflammation, compared with baseline values. Overall, these findings suggest that genistein-containing soy isoflavones may modulate eosinophil-driven inflammatory pathways in asthma. However, the interpretation of these results is limited by the small sample size, short duration of intervention, and the use of mixed isoflavone preparations, which preclude definitive conclusions regarding clinical efficacy (75). To determine whether mechanistic and early translational findings translate into clinical benefit, larger randomized controlled trials have evaluated soy isoflavone supplementation in asthma. In a multicenter, randomized, double-blind, placebo-controlled study, soy isoflavone supplementation (100 mg/day for 24 weeks) was administered to adolescents and adults with poorly controlled asthma. The trial enrolled 386 participants and assessed lung function, symptom burden, asthma control scores, and inflammatory biomarkers. After 24 weeks of intervention, no significant differences were observed between the soy isoflavone and placebo groups in forced expiratory volume in 1 second (FEV1), symptom scores, frequency of poor asthma control episodes, or fractional exhaled nitric oxide levels. Although plasma genistein concentrations increased significantly in the intervention group, these pharmacokinetic changes did not translate into measurable improvements in clinical outcomes or airway inflammation (76).

#### 5.2.5 Chronic kidney disease

A pilot randomized, double-blind, controlled study evaluated the effects of soy isoflavone supplementation in patients with end-stage renal disease undergoing chronic hemodialysis, a population characterized by chronic kidney disease and elevated systemic inflammation (CRP >10 mg/L). Participants received either isoflavone-containing soy-based nutritional supplements or isoflavone-free milk protein for 8 weeks. Soy isoflavone supplementation resulted in a 5-10-fold increase in circulating isoflavone levels compared with controls, confirming adequate systemic exposure. However, at the group level, no statistically significant differences were observed in inflammatory markers after the intervention, although a non-significant trend toward lower CRP levels was noted in the soy-supplemented group. Interestingly, individual changes in isoflavone concentrations showed an inverse correlation with changes in CRP and a positive correlation with nutritional status markers, including albumin and insulin-like growth factor-1 (IGF-1). These observations suggest a possible association between soy isoflavone exposure and modulation of inflammatory and nutritional parameters in hemodialysis patients. Nevertheless, the interpretation of these findings is limited by the small sample size, short study duration, and substantial inter-individual variability, and they do not allow firm conclusions regarding clinical efficacy (77).

#### 5.2.6 Psoriasis

Psoriasis is a chronic inflammatory skin disease driven by immune dysregulation and keratinocyte hyperproliferation, leading to persistent cutaneous inflammation; while it is commonly considered an autoimmune condition, this classification is not universally accepted, and thus psoriasis is often more broadly included among immune-mediated inflammatory diseases (68, 78). A randomized, double-blind, placebo-controlled trial in patients with mild-to-moderate chronic plaque psoriasis evaluated genistein administered at doses of 75 mg/day and 150 mg/day over 56 days. Overall, no statistically significant differences were observed between the genistein and placebo groups in key clinical endpoints, including the Psoriasis Area and Severity Index (PASI), Body Surface Area (BSA), or Physician’s Global Assessment (PGA). A borderline effect was noted for PGA at day 56 (p = 0.0506), but this did not meet conventional thresholds for statistical significance, and thus overall clinical efficacy was not demonstrated. In an exploratory subgroup analysis, however; marked inter-individual variability in response was observed, with isolated patients showing reductions in PASI scores and partial lesion improvement (79).

Overall, randomized controlled trials suggest that genistein may exert modest beneficial effects on metabolic and inflammatory biomarkers across several chronic disease settings. However, these effects are heterogeneous, and robust disease-modifying efficacy has not been demonstrated. Importantly, evidence in autoimmune diseases remains limited, with only isolated clinical data available in Hashimoto’s thyroiditis, while immune-mediated inflammatory diseases, such as psoriasis, show predominantly mechanistic or exploratory signals without consistent clinical benefit in controlled trials.

## 6 Context-dependent immunomodulatory effects of genistein

One of the key challenges in interpreting the immunological effects of genistein is the marked context dependency of its biological activity. Although often described as an anti-inflammatory or immunosuppressive compound, available evidence indicates that its effects depend on disease context, dose, timing of administration, hormonal milieu, sex, and the specific immune pathways involved. Accordingly, genistein should be regarded not as a uniformly immunosuppressive agent, but as a pleiotropic immunomodulator capable of exerting both protective and potentially adverse effects under different conditions. This complexity is well illustrated in experimental models of T1D. In adult NOD mice, genistein delayed disease onset, reduced incidence, and preserved pancreatic islet architecture, indicating a protective immunomodulatory effect. However, in perinatal exposure models, particularly in female offspring, it increased susceptibility to T1D and promoted a more pro-inflammatory immune profile associated with alterations in gut microbiota composition. These findings highlight the importance of developmental stage, sex, hormonal environment, and microbiota-immune interactions in shaping outcomes (56–59).

Dose dependency has also been reported in autoimmune ovarian disease, where higher doses of genistein improved ovarian histology and reduced inflammation, whereas lower doses exacerbated disease manifestations. This suggests biphasic, non-linear effects potentially linked to its phytoestrogenic properties and differential activation of estrogen receptor-dependent pathways (63).

Timing of intervention is another critical determinant. In experimental EAE, early administration of genistein reduced neuroinflammation and demyelination while promoting regulatory responses, including increased Foxp3^+^ regulatory T-cell (Treg) populations and IL-10 production. In contrast, treatment initiated after disease establishment showed limited efficacy and failed to reverse established neurological deficits, indicating greater effectiveness during early inflammatory phases (54, 55).

A particularly clear example of context dependency is observed in Treg regulation. While the above-mentioned observations indicate the immunosuppressive effects of genistein, in tumor microenvironment models, genistein suppresses Treg differentiation and function, thereby enhancing CD8^+^ T-cell-mediated antitumor immunity and improving responses to immune checkpoint blockade (41). Importantly, the immunological effects of genistein are not uniformly suppressive and vary depending on the dominant immune pathways in a given disease setting. Across several autoimmune and inflammatory models, it enhances regulatory mechanisms while attenuating pro-inflammatory cytokine production. However, its interactions with estrogen receptors, tyrosine kinases, oxidative stress pathways, and microbiota-immune signaling networks suggest a broader immunomodulatory profile that may not consistently translate into beneficial outcomes, particularly in diseases with heterogeneous immune phenotypes.

Collectively, the available evidence supports the view that genistein functions as a context-dependent immunomodulator rather than a classical broad-spectrum immunosuppressive agent. Its effects vary with disease stage, immune phenotype, dose, sex, endocrine status, dietary background, and tissue-specific signaling pathways. Recognition of this complexity is essential for correct interpretation of preclinical data and for the rational design of future clinical studies in autoimmune and immune-mediated diseases.

### 6.1 Sex as a biological variable in genistein-mediated immunomodulation

Sex represents a critical and often underappreciated determinant of genistein’s immunological effects, acting in close interplay with its phytoestrogenic properties and estrogen receptor-mediated signaling. The preclinical evidence summarized above strongly supports sex-dependent variability in both disease susceptibility and treatment outcomes across autoimmune disease models. This is particularly evident in type 1 diabetes models, where perinatal genistein exposure increased disease susceptibility in female offspring while inducing distinct immunological and microbiota-associated changes in males. These divergent outcomes suggest that early-life exposure to phytoestrogens may differentially program immune trajectories in a sex-dependent manner, potentially through long-term modulation of endocrine-immune-microbiome interactions. Additional support for sex-dependent effects arises from autoimmune ovarian disease models, in which genistein exerts dose-dependent estrogen-like activity directly influencing ovarian physiology and immune-mediated tissue damage. Moreover, the involvement of estrogen receptor signaling pathways (including ERα and ERβ) in other autoimmune contexts, such as Sjögren’s syndrome and systemic lupus erythematosus, further underscores the importance of hormonal milieu in shaping genistein’s biological effects.

Taken together, these findings indicate that sex and hormonal status are not merely modifying variables but represent fundamental determinants of genistein activity. This is particularly relevant given the female predominance observed in many autoimmune diseases, including systemic lupus erythematosus, Sjögren’s syndrome, and autoimmune thyroid disease. Therefore, sex-specific immune and endocrine interactions should be considered an essential component in the interpretation of both preclinical results and future clinical translation of genistein-based interventions.

## 7 Strengths and limitations of current evidence

The available evidence suggests that genistein exerts broad immunomodulatory and anti-inflammatory effects across multiple autoimmune and immune-mediated disease models. One of the major strengths of the current literature is the consistency of several mechanistic observations across diverse experimental systems. In both *in vitro* and *in vivo* studies, genistein has repeatedly been shown to modulate key inflammatory pathways, including NF-κB, MAPK, STAT, oxidative stress-related signaling, and cytokine production. In addition, several studies demonstrated effects on immune cell polarization, T-cell responses, and epithelial or tissue-specific inflammatory processes, supporting the concept that genistein acts as a multi-target immunomodulatory compound rather than through a single molecular mechanism. Another important strength is the breadth of disease models investigated. Genistein has been evaluated in systemic autoimmune diseases such as systemic lupus erythematosus and rheumatoid arthritis, neuroinflammatory conditions including experimental autoimmune encephalomyelitis, endocrine autoimmune disorders such as type 1 diabetes, as well as autoimmune blistering skin diseases and psoriasis-like inflammation. The observation of partially convergent anti-inflammatory effects across these distinct pathological contexts may support the biological plausibility of its immunoregulatory activity. Several studies also provide relatively detailed mechanistic insights. Particularly in rheumatoid arthritis, pemphigus vulgaris, Sjögren’s syndrome, and experimental autoimmune encephalomyelitis, the molecular pathways affected by genistein have been investigated extensively, including effects on ROS/Akt/NF-κB signaling, ferroptosis-related pathways, cytokine networks, endocytic trafficking, and regulatory T-cell responses. Furthermore, some experimental studies demonstrated histopathological improvement and reduction of disease severity *in vivo*, supporting the translational relevance of these observations.

Despite these promising findings, the current evidence base has substantial limitations. Most importantly, many available data derive from *in vitro* experiments and animal models, whereas clinical evidence remains extremely limited. Only a single randomized controlled trial has specifically evaluated genistein in a classical autoimmune disease, namely Hashimoto’s thyroiditis. Consequently, the translational applicability of many preclinical findings to human autoimmune disease remains uncertain. Considerable heterogeneity also exists across experimental studies. Differences in animal models, dosing regimens, treatment duration, formulation, timing of administration, and dietary background substantially complicate direct comparisons between studies. In several disease models, including type 1 diabetes and autoimmune ovarian disease, genistein exhibited dose-dependent or sex-dependent effects, with some studies reporting potentially adverse or paradoxical immune responses under specific experimental conditions. These observations suggest that the immunological effects of genistein may be context-dependent and influenced by hormonal status, microbiota composition, or concomitant phytoestrogen exposure. Another important limitation is the relatively limited reproducibility and independent validation of several findings. Many mechanistic observations originate from single research groups and have not yet been replicated extensively in independent experimental settings. In addition, some disease areas remain supported only by preliminary or exploratory evidence. Importantly, not all clinical studies demonstrated beneficial effects. While some randomized controlled trials reported improvements in inflammatory or metabolic biomarkers, larger studies in asthma and psoriasis failed to demonstrate significant clinical efficacy despite measurable biological exposure to genistein. These discrepancies indicate that modulation of inflammatory pathways does not necessarily translate into meaningful clinical improvement and underscore the complexity of immune-mediated diseases in humans. Finally, many clinical studies were limited by relatively small sample sizes, short intervention periods, heterogeneous patient populations, and the use of mixed soy isoflavone preparations rather than purified genistein alone. Therefore, the currently available clinical evidence remains insufficient to establish genistein as an evidence-based therapeutic strategy in autoimmune diseases.

Overall, the existing literature supports the biological and immunomodulatory potential of genistein; however, the strength of evidence varies considerably between disease models and remains predominantly preclinical. Further well-designed mechanistic studies and adequately powered randomized clinical trials are necessary to determine its therapeutic relevance, optimal dosing strategies, long-term safety, and disease-specific efficacy in human autoimmune disorders.

## 8 Translational considerations

### 8.1 Bioavailability

Despite its broad pharmacological activity, genistein faces major barriers to clinical translation, primarily due to an unfavorable pharmacokinetic profile. Low aqueous solubility, extensive first-pass metabolism, and rapid clearance limit its oral bioavailability and therapeutic plasma levels. Moreover, rapid conjugation into glucuronide and sulfate forms further reduces the availability of the active compound, hindering the translation of its biological effects into clinical outcomes. Given its ability to modulate key cancer-related processes - including apoptosis, autophagy, angiogenesis, metastasis, immune response, and cell cycle progression - improving genistein delivery has become a central focus in anticancer research. Nanotechnology-based delivery systems offer a promising strategy to overcome these limitations. Formulations such as polymeric nanoparticles, liposomes, solid lipid nanoparticles, and nanoemulsions enhance solubility, stability, and resistance to metabolic degradation, thereby improving absorption, circulation time, and intracellular accumulation. Furthermore, functionalized nanocarriers enable more targeted delivery through ligand-mediated uptake (e.g., antibodies, peptides, folate), increasing selectivity toward cancer cells while reducing off-target toxicity. Controlled and sustained release from these systems prolongs tumor exposure to genistein, enhancing its effects on oncogenic pathways and tumor inhibition. Preclinical studies report encouraging results, particularly in breast, lung, and colon cancers. Overall, nanocarrier-based strategies may bridge the gap between genistein’s strong preclinical potential and its limited clinical applicability by improving bioavailability, targeting efficiency, and safety, while also supporting better patient compliance (80, 81).

### 8.2 Safety, side effects, and drug interactions

Genistein is a soy-derived isoflavone with structural similarity to estrogens and a long history of dietary exposure. Its biological effects are not fixed but depend on context-particularly dose, metabolic factors, and individual variability. Evidence from experimental, clinical, and epidemiological studies suggests a dual profile: genistein may confer benefits in hormone-related conditions and disease prevention, yet under certain exposure conditions it can also produce less favorable effects. This apparent contradiction reflects its interaction with estrogen signaling pathways, where it can act as a weak modulator at physiological levels but exhibit more pronounced endocrine activity at higher doses, highlighting a context-dependent balance between potential benefit and risk (82). This complexity is mirrored in toxicological data. In studies on Wistar rats exposed to genistein over acute, subchronic, and long-term (52-week) periods, low to moderate doses were generally well tolerated. At higher doses (500 mg/kg/day), effects such as reduced food intake, lower body weight gain, mild hematological changes, altered organ weights, and reversible histological findings in organs including the reproductive system, liver, kidney, and spleen were observed. Importantly, these effects were largely interpreted as manifestations of estrogen-like pharmacological activity rather than overt toxicity. Most changes were reversible, and only mild hepatic and biochemical alterations persisted at the highest dose. On this basis, the no observed adverse effect level (NOAEL) was established at 50 mg/kg/day, and the no observed effect level (NOEL) at 5 mg/kg/day (83).

From a mechanistic standpoint, genistein is also recognized as a naturally occurring inhibitor of tyrosine-specific protein kinases (10). While inhibition of these enzymes is a well-established strategy in oncology, it has also been linked to immune-related adverse effects, including autoimmune phenomena. Although such effects are relatively uncommon, they are clinically relevant. Proposed mechanisms include disruption of immune homeostasis, altered signaling within immune cell populations, impaired tolerance, and dysregulated inflammatory responses - all consistent with the central role of tyrosine kinases in immune regulation (84). In this context, genistein acts as a relatively weak and non-selective inhibitor, which may partly account for its broad, pleiotropic biological activity.

Beyond its effects on signaling pathways, genistein can also influence drug metabolism and transport. *In vitro* studies show that it inhibits several cytochrome P450 enzymes, particularly CYP2C9 and CYP3A4, via noncompetitive mechanisms. However, despite measurable inhibition (with Ki values in the micromolar range), pharmacokinetic data indicate that typical systemic concentrations are generally too low to produce clinically meaningful interactions *in vivo*, suggesting a limited risk of significant drug–drug interactions under normal exposure conditions (85, 86).

Additional interactions involve membrane transport systems. Genistein has been shown to modulate ATP-binding cassette (ABC) transporters, including P-glycoprotein (ABCB1), MRPs (ABCC family), and ABCG2, which play key roles in drug absorption, distribution, elimination, and multidrug resistance (87). More broadly, dietary isoflavones may influence both enzyme activity and transporter expression, potentially altering the pharmacokinetics of co-administered drugs - particularly in the context of supplementation or high intake (88).

## 9 Conclusion

Genistein is a multifunctional dietary isoflavone that links endocrine signaling with kinase-related and immunoregulatory pathways, resulting in broad (yet context-dependent) biological activity in models of autoimmune and inflammatory diseases. Its effects vary with dose, physiological context, and hormonal environment, which complicates straightforward interpretation. Preclinical studies consistently show that genistein can influence immune cell function, inflammatory cascades, and tissue-specific pathology. However, these effects are not uniform; depending on the model and exposure conditions, they may be bidirectional or stage dependent. This variability underscores the difficulty of translating mechanistic findings into predictable therapeutic outcomes. Clinical evidence remains limited and heterogeneous. Importantly, most clinical studies conducted to date have focused on metabolic or chronic inflammatory conditions rather than classical autoimmune diseases. Clear disease-modifying efficacy in autoimmune conditions has not been established. To date, only one randomized controlled trial has specifically evaluated genistein in an autoimmune disease setting, namely Hashimoto’s thyroiditis; therefore, clinical efficacy in autoimmune diseases remains largely unproven. Preliminary controlled data in autoimmune thyroid disease suggest immunoendocrine modulation, but confirmation across other autoimmune disorders is still lacking. From a translational standpoint, genistein illustrates both the promise and the challenges of pleiotropic natural compounds. Its multitarget activity may be advantageous in complex disease networks, yet the same lack of specificity - combined with variability in bioavailability and metabolism - limits clinical predictability. Future research should focus on standardized dosing, detailed pharmacokinetic profiling, and well-designed, disease-specific clinical trials. Defining exposure-response relationships will be critical to determine whether genistein can progress from a bioactive dietary compound to a clinically relevant adjunct in the management of autoimmune and inflammatory diseases. Importantly, based on available toxicological and clinical data, genistein demonstrates a relatively favorable safety profile at typical exposure levels. Even if future clinical trials confirm limited or clinically non-significant efficacy, the risk of persistent or severe adverse effects appears low. Reported effects are generally mild, dose-dependent, and reversible, supporting the view that genistein - within studied ranges - poses minimal long-term safety concerns.
